# Correction to “Analysis of factors influencing the recurrence of diabetic foot ulcers ”

**DOI:** 10.1111/srt.70032

**Published:** 2024-09-10

Hu L, Liu W, Yin L, Yi X, Zou Y, Sheng X. Analysis of factors influencing the recurrence of diabetic foot ulcers. Skin Res Technol. 2024;30:e13826. https://doi.org/10.1111/srt.13826

In page 1 the platform: Department of Endocrinology, The Third Affiliated Hospital of Nanchang University (The First Hospital of Nanchang), Nanchang, Jiangxi, China, is incomplete. Complete platform is: Department of Endocrinology, The Third Affiliated Hospital of Nanchang University (The First Hospital of Nanchang), Jiangxi Provincial Key Laboratory of Metabolical Endocrinology (2024SSY06121), Nanchang, Jiangxi, China.

In this paper no funding is incorrect. Please add funding on page 6: Funding: This research is funded by the Key research and development projects of Jiangxi Province (No. 20203BBG73053).

We apologize for this error.

This is an open access article under the terms of the Creative Commons Attribution License, which permits use, distribution and reproduction in any medium, provided the original work is properly cited.

© 2024 The Authors. *Skin Research and Technology* published by John Wiley & Sons Ltd.

Skin Res Technol. 2024;30:e13852

https://doi.org/10.1111/srt.13852

wileyonlinelibrary.com/journal/srt

